# Perceived causes and management of epilepsy among rural community dwellers in Ghana: a qualitative synthesis

**DOI:** 10.3389/fneur.2023.1230336

**Published:** 2023-10-04

**Authors:** Daniel Gyaase, Theresah Ivy Gyaase, Rebecca Tawiah, Godfred Atta-Osei, Isaac Owusu, Wisdom Kwadwo Mprah, Yeetey Akpe Enuameh

**Affiliations:** ^1^Department of Epidemiology and Biostatistics, School of Public Health, Kwame University of Science and Technology, Kumasi, Ghana; ^2^Center for Disability and Rehabilitation Studies, Kwame University of Science and Technology, Kumasi, Ghana; ^3^Department of Health Promotion and Disability Studies, School of Public Health, Kwame University of Science and Technology, Kumasi, Ghana

**Keywords:** epilepsy, perceived causes, management, seizures, epileptic

## Abstract

**Background:**

In Ghana, over 270,000 people live with epilepsy, of which 70% do not receive treatment. Despite the high number of people with the condition, misconceptions exist about its causes and management in African regions. The study assessed the perceived causes and management of epilepsy among rural community dwellers in Ghana.

**Methods:**

A qualitative approach and phenomenological design were employed for the study. The population comprised community dwellers in Berekum, a rural town in the Bono Region of Ghana. A convenience sampling technique was used to sample the participants. An in-depth face-to-face interview with a semi-structured interview guide was used to collect participant data. Data were analyzed using inductive thematic analysis.

**Result:**

A total of 15 participants were interviewed in the study, after which saturation was reached. Seven of the participants were men, and eight were women. Two categories emerged as the causes of epilepsy: socio-cultural and superstitious causes and biomedical causes. The socio-cultural and superstitious causes include “a manifestation or an influence of an evil spirit,” “family curse or disease,” “punishment from ancestors or gods of the land,” “having several convulsions,” “exposure to foam from an epileptic,” and “bites from an epileptic during seizures”, while the biomedical causes are “brain damage,” “blood group,” and “genetic makeup”. Consulting with the spiritual realm, pouring water on the person or washing the person's face, and putting a spoon in the mouth were identified by the participants as ways to manage epilepsy.

**Conclusion:**

The causes of epilepsy are primarily linked to the supernatural, with the results indicating that rural community residents largely attribute epilepsy to “evil spirits”. This implies that the rural communities' knowledge about the causes of epilepsy is based on the social causation theory of disease and disability, which relates diseases to the supernatural. Management of the condition was mainly seen as spiritual.

## Introduction

Epilepsy is a chronic brain disorder characterized by recurrent nervous system disturbance resulting from a sudden excessive disorderly discharge of the aggregate group of neurons from the cerebrum, leading to disturbances of sensation, motor signals and symptoms, or psychiatric and behavioral aspects with or without loss of consciousness (1). The World Health Organization (WHO) defines it as a chronic brain disease characterized by recurrent seizures with brief episodes of involuntary movement with either a part or the entire body and sometimes accompanied by loss of consciousness and control of bladder function (2).

Epilepsy is reported as the most common neurological condition, with an estimated 50 million people across the globe living with it. A global estimate shows that ~80% of those with the condition live in low- and middle-income countries (2), with less or no access to medical services or treatment (3). In Africa, an estimated 10 million people are living with epilepsy (4), with 5.4 million people in sub-Saharan Africa living with lifetime epilepsy (5). In Ghana, it is reported that 270,000 people are living with epilepsy, of whom 70% do not receive treatment (6).

Despite the high number of people with the condition, misconceptions exist about its causes and management in African regions. In ancient times, when people had no understanding or knowledge of biomedical science, epilepsy was attributed to spiritual or religious afflictions (4, 7), and its management was to seek care from traditional healers and people with spiritual powers (4). Epilepsy is a widely recognized health condition, but it is also poorly understood, even by persons close to the persons with it. Lack of knowledge about its causes has led to negative perceptions and beliefs, poor treatment, and stigma associated with it (8). According to Al-Dossari et al., the initial step toward devising strategies to dispel myths and misconceptions about the disorder involves assessing the knowledge, comprehension, beliefs, and causes associated with the condition (9).

Kassie et al. revealed a knowledge gap regarding the causes, nature, and treatment of epilepsy with varying traditional beliefs, attitudes, and practices worldwide (10). In Africa, the knowledge of epilepsy is fused into cultural and religious practices (11). People living with epilepsy and their families are faced with economic, social, and physical (injuries) challenges, mainly due to the stigma and misconceptions of the disorder (4). Individuals with the disorder are discriminated against in education, marriage, driving, and employment (4, 12). Understanding people's perspectives on the causes of epilepsy and its treatment is relevant to reducing the large treatment gap in managing the condition in Africa (11). Since the passage of Ghana's Disability Law (715) and subsequent ratification of the Convention on the Rights of Persons with Disabilities, attempts have been made to change people's perceptions and attitudes toward persons with disabilities, including those with epilepsy, but much has not been achieved generally. Most studies in Ghana focused on conditions such as hearing loss, visual impairment, physical disabilities, and autism. There is less information on the perception of the causes of epilepsy and how this influences its management. This study, therefore, sought to assess the perceived causes and management of epilepsy among rural community dwellers in Ghana.

## Methods

The consolidated criteria guide for reporting qualitative research (COREQ) checklist (13) was used in reporting the study.

### Study design

The methodological orientation employed in this study was phenomenological design. The population comprised community dwellers in Berekum, a town in the Bono Region of Ghana. The inclusion criteria were people who are from the community or lived the community for more than 15 years and aged above 18 years.

### Sampling technique and sample size

A convenient sampling technique was used to sample the participants. Participants were recruited conveniently around roadsides, marketplaces, lorry stations, and churches. The purpose of recruiting participants from the area was to generate evidence on how epilepsy is perceived in the community. Potential participants were approached by the researcher face-to-face and invited to participate in the study. In all, 15 participants were recruited for this study because the saturation point was reached after interviewing this number.

### Data collection

An in-depth face-to-face interview with a semi-structured interview guide was used to collect participant data. The guide was developed by D.G. based on the objectives of the study and revised by Y.E., the supervisor. Questions were open-ended; however, probes and follow-ups were utilized when needed. All interviews were conducted by D.G. in Akan (Twi) for 2 weeks in March 2017. Field notes and audio recordings were taken during the interviews. The duration of interviews was between 30 and 40 min. The recorded interviews were transcribed by D.G. in Twi verbatim and translated into English, which were then sent to other co-authors (T.I.G., R.T., and G.A.O.) for quality checks. D.G. and G.A.O. are Akan with Twi as their first language (speaking and writing).

### Data analysis

Data were analyzed using inductive thematic analysis. Notes taken during the interviews were processed together with the audio transcriptions. Data were studied carefully, and themes were developed based on participants' responses to questions. Two authors conducted the thematic analysis and coding which were reviewed and discussed by all study authors.

## Results

### Demographic characteristics of the study participants

Table 1 shows the demographic characteristics of the participants. A total of 15 participants were involved in the study, of whom 7 were men and 8 were women. Regarding their age, seven of the participants were in the age group of 40–49 years. With employment status, eight were self-employed, four were employed, and three were students. In all, 13 participants have had some form of education.

**Table 1:** Demographic characteristics of participants.

**Variables**	**Frequency (*N* = 15)**	**Percentage (%)**
**Age groups (years)**		
20–29	2	13.3
30–39	4	26.7
40–49	7	46.7
50–59	2	13.3
**Sex**		
Male	7	46.7
Female	8	53.3
**Employment status**		
Employed	4	26.7
Self-employed	8	53.3
Student	3	20
**Educational status**		
No formal education	2	13.3
Basic	7	46.7
SHS	2	13.3
Tertiary	4	26.7

SHS, Senior High School.

### Perceived causes of epilepsy

From participants' narratives, two primary themes, each with subthemes, emerged regarding the causes of epilepsy. These encompass (a) socio-cultural and superstitious factors and (b) biomedical factors. The socio-cultural and superstitious aspects encompass notions of “manifestation or influence of an evil spirit,” “family curse or disease,” “punishment from ancestors or gods of the land,” “frequent convulsions,” “exposure to an epileptic's foam,” and “bites from an epileptic during seizures”. Conversely, the biomedical causes consist of “brain damage,” “blood group,” and “genetic composition”.

### Socio-cultural and superstitious causes

#### A manifestation or influence of an evil spirit

As per the study's findings, epilepsy was perceived as a representation of an “*evil spirit's*” presence in a person's life, suggesting a connection where the spirit either possesses the individual or the person maintains a relationship with malevolent forces. For example, according to a participant,

“*When a person or an individual is possessed by an evil spirit, the spirit manifests itself as epilepsy… therefore people get to know that this individual is possessed by an evil spirit”* (Participant 2).

Some participants, however, thought that epilepsy is not just about being possessed by the “evil spirit” but a sign of being burdened by an “evil spirit”. This manifests as epilepsy if the person can no longer bear the burden. A participant explained this in the following quotation:

“*When there is a spiritual burden on someone, it manifests as epilepsy. Thus, when the person is tired in the spiritual realm or is possessed with an evil spirit, it manifests physically in the form of epilepsy”* (Participant 4).

Some of the participants believed that epilepsy is used to sabotage individuals who are perceived as having a bright future, usually by a witch family member, as exemplified in the following quotation:

“*Epilepsy is a condition that someone gives to another person through spiritual means when they see a bright future ahead of them. It only affects people when the witches in their family notice that the person has a bright future and through spiritual means, they place it on that individual”* (Participant 5).

Another participant added,

“*I know that epilepsy is caused by evil spirits. When an evil spirit in a family sees the bright future of a person, then they buy it [epilepsy] in the spiritual realm for the person”* (Participant 3).

#### Family curse

Another socio-cultural notion among the participants concerning the cause of epilepsy is the idea that the condition is restricted to specific cursed families. Consequently, individuals are thought to not merely acquire epilepsy by chance but rather inherit it as a familial or bloodline curse. To them, some families are cursed to give birth to individuals with epilepsy, as stated by a participant,

“*Epilepsy is a family disease or the result of a curse in a family that manifests within family members”* (Participant 5).

Another respondent added,

“*In some families, there are curses placed on them, and the sign of the curse is epilepsy. Hence members of the family become sufferers of it”* (Participant 10).

#### Punishment from ancestors or gods of the land

The study uncovered that when an individual commits a transgression (engages in misconduct, commits crimes, performs evil actions, or acts immorally) against the deities or forebears of a community, the wrath and displeasure of these entities descend upon the person, and the consequence they face is the onset of epilepsy. Epilepsy, according to this belief, is acquired.

“*When a person sins against the ancestors or gods of the land, the anger of the gods comes upon that person. The person is being punished by the gods by making him or her epileptic”* (Participant 8).

Similarly,

“*People commit some immoral acts which are against the tractions of this land, and a way of punishment the wrap of the gods comes on them, and one of wrap that comes is epilepsy”* (Participant 14).

#### Experiencing several convulsions at a younger age

Participants also perceived that if an individual experiences numerous convulsions during their childhood and proper measures are not taken to address them, it could lead to the development of epilepsy. According to participants, certain parents underestimate the gravity of convulsions and assume that the child will naturally recover over time. Consequently, these parents do not seek timely medical attention for their child, resulting in the child eventually growing up with epilepsy. A participant explained:

“*When a child experiences several convulsions, and the family is unable to seek healthcare for the child, it leads to epilepsy when he or she grows up”* (Participant 1).

Some participants attributed frequent convulsions to excess phlegm in a person's body, which, if not cared for adequately, could later result in epilepsy. A participant noted:

“*If phlegm's become much in a child's system, the child begins to experience a convulsion and if the family do not pay much attention, it leads to epilepsy”* (Participant 1). *[SIC]*

#### Exposure to epileptic foam and bites

As per the accounts of certain participants, it is perceived that exposure to the foam produced from the mouth of an individual experiencing an epileptic seizure could lead to the development of the condition. Consequently, people take precautions to avoid contact with the foam in order to prevent acquiring the condition themselves. As explained by a participant,

“*During epileptic seizures, when the foam comes in contact with another person, that person also develops epilepsy”* (Participant 9).

Furthermore, participants explained that one could become epileptic when a person with epilepsy bites them:

“*When an epileptic patient bite you during epileptic seizures, you will have epilepsy”* (Participant 3). *[SIC]*

### Biomedical causes

#### Brain damage

Some participants associated epilepsy with biomedical factors, including brain damage resulting from accidents as well as incidents occurring during pregnancy and childbirth. According to this viewpoint, when an accident occurs and an individual experiences a brain injury, the person may develop epilepsy. It has been noted that when there is any injury to a child's brain during pregnancy or childbirth, the child may develop epilepsy after birth. Two participants explained this in the following quotations:

“*Epilepsy can be due to brain damage resulting from an accident. When someone has epilepsy, it means that the person is suffering from brain damage, and during the seizures, the person becomes unconscious”* (Participant 7).

“*Most women are careless during pregnancy, and they end up damaging the child's brain whiles in the womb. Also, during delivery, some complications can arise which can cause damage to the brain and later leads to epilepsy”* (Participant 3). *[SIC]*

#### Blood group and genetic makeup

Some other participants linked the cause of epilepsy to the compatibility of the parent's blood groups. It was noted by participants that some blood groups are not compatible. Hence, when these individuals marry and give birth, the child is likely to develop complications such as epilepsy. A participant explained:

“*There is some blood that is not compatible with others, and so when a couple's blood group are not compatible, and they give birth, the child they will give birth to may develop epilepsy”* (Participant 3).

Additionally, some participants reported that epilepsy is genetic; hence, one may acquire the condition if either of the parents has it.

“*When a parent has epilepsy, genetically their child too can acquire it”* (Participants 3 and 12).

#### Perceived management of epilepsy

The following themes emerged in the community's management of epilepsy: “consultation from the spiritual realm,” “pouring water on the person or washing the person's face,” and “putting a spoon in the mouth”.

#### Consultation from the “spiritual realm”

The study's findings revealed that epilepsy is interpreted as a spiritual affliction that impacts individuals. Participants hold the view that this ailment or state cannot be remedied through medical interventions but rather requires spiritual remedies. Consequently, seeking counsel from individuals possessing spiritual abilities, such as fetish priests, witch doctors, mallams (Muslim ritualists in Ghana), pastors, and similar practitioners, is perceived as the most effective approach for treating the condition. Several of the participants explained as follows:

“*Epilepsy is a spiritual disease that affects people … there is no physical cure or medication that can cure it … but it can be cured through spiritual means.”*

#### Pouring water on the person or washing the person's face

Some participants disclosed that they would splash water on the individual or cleanse their face in order to restore their consciousness, as indicated by one participant:

“*When I see one, I will look for water and wash the person's face till he or she comes to consciousness”* (Participant 3).

Participants also noted that dousing a sleeping person with water leads to their awakening, similarly, pouring water on an unconscious individual causes them to regain consciousness. A participant elaborated:

“*I will pour water on the person when the seizures come. This is to bring the person back to consciousness. I know that when one is asleep, and you pour water on the person or wash the person's face with water, the person wakes up from sleep, and I think when that is also done to someone who is in a seizure, the person will come back to consciousness”* (Participant 8).

### Putting a spoon in the mouth

Some participants also reported that another way to prevent a person with epilepsy from biting the tongue is to insert a spoon in the person's mouth.

“*I will put a spoon in the person's mouth to prevent him or her from swallowing or biting the tongue”* (Participant 15).

## Discussion

This study was conducted in rural communities in Ghana to assess the perceived causes and management of epilepsy. Epilepsy is one of the major chronic non-communicable diseases of the brain in Ghana, with ~270,000 people living with the condition (6). It is the most prevalent neuropsychiatric condition reported in rural health institutions and is among the top five medical conditions in the country. However, there is a treatment gap, with only ~15% of people with epilepsy receiving treatment and care (6). This study is therefore relevant, as it has highlighted perceptions about the causes of epilepsy in a rural setting that affect the management and care of people with epilepsy in such settings.

The finding that evil spirits cause epilepsy is not new, but this old-age misconception has become deep-seated in Ghana, especially in rural areas. The findings from this study seem to reinforce this misconception about the cause of epilepsy. This finding is similar to Abasiubong et al.'s study, which revealed that in the olden days when people had no knowledge or understanding of biomedical science, epilepsy was attributed to a spiritual or religious affliction (14). Even though there is advancement in biomedical science at this present time, people still have ancient beliefs about epilepsy as the manifestation of an evil spirit in the victim. Awareness about epilepsy is needed, especially in rural settings, to manage the condition associated with secondary outcomes, such as burns and falls.

Studies have revealed that what people perceive most to be the cause of epilepsy among rural dwellers is witchcraft. In research led by Abasiubong et al. regarding how traditional healers perceive the causes of epilepsy, witchcraft stood out as the prevailing factor (14). In the present study, the community members' perception of the cause of epilepsy aligns with that of the aforementioned study. The community dwellers believe that witches can see into the future; therefore, when they foresee a person's future in the family is bright and they do not want that to happen, through witchcraft, they buy the sickness and place it on the person's life. As the individual continues to have seizures, people will isolate themselves from the person, and that future will not come to pass.

The study findings show that the social causation theory of disease and disability is dominant in rural areas. According to Krieger, the theory views sickness and disease as the consequences of disintegration at the social level through anti-social behavior and supernatural intervention or possession (15). Concerning the Akans, most neurological and mental disorders are attributed to witchcraft (15). People's supernatural beliefs, such as witchcraft and punishment from the gods, are frequently attributed as the cause of neurological disorders like epilepsy.

Epilepsy is perceived as a familial affliction or a generational curse that casts its shadow upon family members. The findings indicate that epilepsy serves as an indicator that a family is under a curse. It is believed that in times past, specific families maintained associations with idols and fetish priests. As these families distanced themselves from idol worship, they allegedly incurred curses, resulting in ailments meant to disgrace the family. Some members of these families now bear the weight of these curses, manifesting as various diseases, including epilepsy. Consequently, epilepsy is viewed as a condition befalling cursed families. Additionally, it is regarded as a sign alerting a particular family to their cursed state, prompting them to seek aid or appeasement from the very idols and fetish priests they once had relations with. Masia and Devinsky stated that epilepsy was seen as a curse for possessing evils, mainly resulting in the isolation of the affected person and the family (16).

The study further reveals punishment from the gods or ancestors for sins committed as a cause of epilepsy. When an individual transgresses or violates the sanctity of the gods, it is held that the gods' wrath befalls that person, resulting in the onset of epilepsy as a form of retribution. This individual is said to experience epilepsy so that the broader community can draw lessons from it. Essentially, epilepsy is considered a means of compelling people to adhere to moral standards. Evidence from a study by Abasiubong et al. (14) justifies the findings of this current study. Their study showed that 14% of the population attributed the cause of epilepsy to punishment for sins (14).

In addition, the study identified brain damage as the cause of epilepsy. Birth complications can result in brain damage, which in turn might lead to the development of epilepsy. Similarly, accidents involving head injuries can also be a factor in causing epilepsy. Consequently, for certain participants, epilepsy is understood as a condition arising from brain damage. Notably, it is reported that 25% of epilepsy cases are preventable; however, a significant proportion of these preventable cases are concentrated in Africa due to elevated rates of prenatal and perinatal complications (4). Even though some participants stated that it was caused by brain damage, they still attributed it to witchcraft. They believe that for an accident to occur, which will involve a person's brain, or for a complication to develop during childbirth, it is all the plans and evil intentions of the witches or gods. Without this, a person's brain will not be damaged. Epilepsy can be genetic or acquired. Epilepsy that is acquired is caused by serious brain trauma, stroke, tumors, or infection in the brain (17).

A bite from an epileptic patient experiencing seizures or exposure to foam during seizures was perceived as a cause of epilepsy. People believe that epilepsy is contagious (18). This notion leads to the understanding that one might contract the condition through bites or contact with the foam produced during seizures. This might explain why community residents fail to guarantee the safety of individuals when they experience seizures in public. In most rural areas and even urban settings in Ghana, people who experience seizure episodes are left alone without anyone ensuring that the surroundings are safe and that the patient will not be hurt. The above-mentioned perceived causes of epilepsy have led to stigma and discrimination toward people with epilepsy. Individuals with the condition are approached with fear and are prevented from participating in social and economic events such as marriage, school, driving, and employment (4).

Convulsions and an abundance of phlegm within the body were considered responsible for the onset of epilepsy. A surplus of phlegm in an individual's system was believed to trigger convulsions, and if these convulsions were not properly addressed during childhood, they could subsequently evolve into epilepsy. Certain parents, who lack an understanding of the severity of convulsions, presume that with the passage of time, the child afflicted by convulsions will naturally overcome them. Consequently, they refrain from seeking prompt medical intervention for their child, which ultimately leads to the child developing epilepsy at a later stage. The findings support Annegers et al.'s study, which found febrile convulsions as a cause of epilepsy (19). Amid convulsions, disruptions within the brain may occur, potentially leading to its impairment and consequently giving rise to epilepsy. A study by Verity and Golding revealed that when young children frequently experience febrile convulsions at an early age, they are more likely to develop epilepsy; that is, if the febrile convulsions occur in the first year of the child's life (20).

Predominantly, epilepsy was perceived as a condition intertwined with “evil spirits”, thereby leading to the approach of seeking guidance from individuals possessing heightened spiritual abilities for its management. A similar finding was discussed in the toolkit “*Advocates' Toolkit for making epilepsy a Priority in Africa*” (4). Due to the misconception and stigma about the condition, most people with epilepsy do not receive medical care. Estimates show that 80% of people with epilepsy in Africa do not receive medical care (4).

## Conclusion

The causes of epilepsy are primarily associated with supernatural factors. The findings indicated that individuals residing in rural communities predominantly attribute epilepsy to “malevolent spirits”. This suggests that the rural population's understanding of epilepsy is rooted in the social causation theory of disease and disability, which connects ailments to supernatural forces. The approach to managing the condition was largely spiritual in nature. Consequently, the study suggests the implementation of comprehensive health education programs concerning neurological and mental health conditions, given their substantial association with supernatural beliefs. Moreover, future investigations should explore the ramifications of these findings on the lived experiences of individuals with epilepsy. Finally, research is warranted to ascertain the prevalence and connections of epilepsy-related injuries.

## Data availability statement

The raw data supporting the conclusions of this article will be made available by the authors, without undue reservation.

## Ethics statement

Approval for the conduct of the study was taken from the Center for Disability and Rehabilitation Study, KNUST. The protocol was reviewed by the Head of Department and the Supervisor of the student work before the study commenced. Written informed consent to participate in the study was from the participants before their involvement.

## Author contributions

DG: conceptualization, data collection, transcription, coding of themes, interpreting the data, writing the original draft, and final review and editing. YE: supervision, coding of themes, and review and editing. TG, RT, GA-O, IO, and WM: review and editing. All authors contributed to the article and approved the submitted version.
